# Network meta‐analysis of randomized trials in multiple myeloma: Efficacy and safety in frontline therapy for patients not eligible for transplant

**DOI:** 10.1002/hon.3041

**Published:** 2022-07-11

**Authors:** Cirino Botta, Emilia Gigliotta, Bruno Paiva, Rita Anselmo, Marco Santoro, Paula Rodriguez Otero, Melania Carlisi, Concetta Conticello, Alessandra Romano, Antonio Giovanni Solimando, Claudio Cerchione, Matteo Da Vià, Niccolò Bolli, Pierpaolo Correale, Francesco Di Raimondo, Massimo Gentile, Jesus San Miguel, Sergio Siragusa

**Affiliations:** ^1^ Department of Health Promotion Mother and Child Care Internal Medicine and Medical Specialties University of Palermo Palermo Italy; ^2^ Clinica Universidad de Navarra CCUN Centro de Investigacion Medica Aplicada (CIMA) IDISNA, CIBERONC Pamplona Spain; ^3^ Division of Hematology Azienda Policlinico‐OVE University of Catania Catania Italy; ^4^ Guido Baccelli Unit of Internal Medicine Department of Biomedical Sciences and Human Oncology (DIMO) School of Medicine Aldo Moro University of Bari Bari Italy; ^5^ Hematology Unit IRCCS Istituto Romagnolo Per Lo Studio Dei Tumori (IRST) “Dino Amadori” Meldola FC Italy; ^6^ Department of Oncology and Hematology‐Oncology University of Milan Milan Italy; ^7^ Hematology Unit Fondazione IRCCS Ca' Granda Ospedale Maggiore Policlinico Milan Italy; ^8^ Medical Oncology Unit Grand Metropolitan Hospital “Bianchi‐Melacrino‐Morelli” Reggio Calabria Italy; ^9^ Hematology Unit Department of Hemato‐Oncology Annunziata Hospital Cosenza Italy

**Keywords:** I line treatment, multiple myeloma, network meta‐analysis, non‐transplant eligible, principal component analysis

## Abstract

The treatment scenario for newly‐diagnosed transplant‐ineligible multiple myeloma patients (NEMM) is quickly evolving. Currently, combinations of proteasome inhibitors and/or immunomodulatory drugs +/− the monoclonal antibody Daratumumab are used for first‐line treatment, even if head‐to‐head comparisons are lacking. To compare efficacy and safety of these regimens, we performed a network meta‐analysis of 27 phase 2/3 randomized trials including a total of 12,935 patients and 23 different schedules. Four efficacy/outcome and one safety indicators were extracted and integrated to obtain (for each treatment) the surface under the cumulative ranking‐curve (SUCRA), a metric used to build a ranking chart. With a mean SUCRA of 83.8 and 80.08 respectively, VMP + Daratumumab (DrVMP) and Rd + Daratumumab (DrRd) reached the top of the chart. However, SUCRA is designed to work for single outcomes. To overcome this limitation, we undertook a dimensionality reduction approach through a principal component analysis, that unbiasedly grouped the 23 regimens into three different subgroups. On the bases of our results, we demonstrated that first line treatment for NEMM should be based on DrRd (most active, but continuous treatment), DrVMP (quite “fixed‐time” treatment), or, alternatively, VRD and that, surprisingly, melphalan as well as Rd doublets still deserve a role in this setting.

## 1 INTRODUCTION

Multiple myeloma (MM) is the second most common hematologic malignancy worldwide.^1^, ^2^ Current milestones of MM therapy include either a quadruple‐, triple‐ or double‐drug combination, based on proteasome inhibitors (PIs) and/or immunomodulatory drugs (IMiDs) plus dexamethasone plus the anti‐CD38 monoclonal antibody (mAb) Daratumumab, with or without chemotherapy. Eligible patients further undergo autologous stem cell transplantation and, eventually, consolidation therapy, while transplant ineligible patients (NEMM) enter follow‐up or maintenance therapy. However, virtually all patients relapse and require further treatments.^1^, ^3^, ^4^, ^5^, ^6^ A plethora of new agents, including second‐generation PIs, histone deacetylase inhibitors, and monoclonal antibodies (mAbs), have shown consistent activity in prospective phase 2/3 clinical trials in relapsed/refractory MM (RRMM) patients and some of them are currently approaching the frontline setting.^4^ In this scenario, current first line treatments for NEMM include the combination of daratumumab + bortezomib, melphalan and prednisone (DrVMP) or lenalidomide and dexamethasone (DrRd) in Europe, while melphalan‐free regimens such as Rd + bortezomib (VRD) or DrRd are the preferred regimens in the USA.^2^ However, the lack of direct head‐to‐head comparisons between approved regimens and the recent introduction of monoclonal antibodies, further complicated the decision‐making regarding frontline strategy for NEMM. To overcome these limitations, we adopted an approach based on network meta‐analysis (NMA) (a recently introduced Bayesian statistical methodology that allows combining direct and indirect evidence to rank the different treatments according to their efficacy and safety^1^, ^5^), to identify regimens with the highest probability of being the most efficacious and safest in this setting.

## 2 METHODS

### 2.1 Search strategy

Relevant publications have been identified through an electronic search of the main relevant databases including PubMed, Embase, Ovid, Cochrane, and proceedings from the major international meetings in hematology and oncology. The following search terms were used: “multiple myeloma”, “Clinical Trials”, “Phase III”, “Phase II”, “Randomized Controlled Trials”, “untreated”, “transplant ineligible”. All titles were screened and selected abstracts were reviewed. The related‐articles function, article references, and Google Scholar were also screened for other applicable publications and were used for searching related studies, abstracts, and citations. Published articles were considered for the analysis if written in English only. The last date of the search was 25 November 2021. A systematic review was performed according to the guidelines and recommendations from the preferred reporting items for systematic reviews and network meta‐analyses (PRISMA) checklist.^7^

### 2.2 Inclusion criteria

Retrieved studies were included into the final analysis if the following criteria were met: (1) they had to involve NEMM (transplant not‐planned); (2) they should be randomized controlled trials, with or without blinding; (3) they could be abstracts, only if they sufficient information on study design, characteristics of participants, interventions, and outcomes were available; (4) they should include patients who received an unconventional or new regimen in the experimental arm, and a standard regimen in the control arm; (5) all trials should have been performed starting from the introduction of the so called “novel agents”: IMiDs and PI.

### 2.3 Exclusion criteria

Studies were excluded from the analysis if they were not comparative, if outcomes of interest were not reported, if the methodology was not clearly reported, if included patients eligible for autologous stem cell transplant (without non‐ASCT subgroup analyses) or relapsed after a frontline therapy.

### 2.4 Data extraction and quality assessment

Three reviewers (C.B., R.A. and E.G.) independently reviewed published literature according to the above predefined strategy and criteria. Each reviewer extracted from each selected study the following data: title and reference details (first author, year), study population characteristics (number of patients in study, number of patients in each treatment), type of interventions, and outcome data. For each trial, we evaluated hazard ratios (HRs) of progression‐free survival (PFS); overall survival (OS); odds ratio (OR) of overall response rate (ORR), complete response (CR); and risk ratio (RR) for safety (evaluation of the most common grade 3–4 toxicity). If the HR of survival curves was not reported, it was derived from the graph by using the method of Tierney et al.^8^ All data were recorded independently in separate databases by all 3 reviewers and were compared just before the final analysis to limit selection bias. The final database was also reviewed an additional investigator (M.S.). Duplicates were removed and any disparity clarified.

All the selected studies were assessed for quality according to the Cochrane Handbook for Systematic Reviews of Interventions, as described elsewhere^1^, ^9^ by computing a score based on the following items (1 point for each of them): method of randomization, allocation concealment, blindness, withdrawal or dropout, and adequacy of follow‐up. Visual inspection of funnel plots were used to assess the presence of publication bias.

### 2.5 Network meta‐analysis

We performed a NMA by using a Bayesian approach to compare the different therapeutic regimens simultaneously. The analysis was performed in STATA software by using the mvmeta package. Network meta‐analysis synthesizes data from a network of trials that involve multiple interventions and therefore, by integrating direct and indirect comparisons, has the potential to rank the treatments according to the outcome. Within the framework of NMA, we ranked the evaluated regimens based on survival outcomes (PFS and OS), treatment efficacy (ORR, CR), and safety (the most frequent grade 3–4 adverse event in each trial). For each outcome, we performed a NMA with an (RE) model by using a Markov chain Monte Carlo simulation technique with up to 30,000 iterations. Loop inconsistency and heterogeneity were assessed by evaluating the log of the ratio of 2 odds ratios (RoR) from direct and indirect evidence in the loop (ifplot command in STATA).^10^, ^11^ RoR values close to 0 indicate that both direct and indirect evidence are in agreement. Heterogeneity of the loop was then assessed through the restricted maximum likelihood method.^10^, ^11^ Relative effects of treatments are reported as HRs for survival outcomes (OS, PFS) and OR or RR for binary outcomes (ORR, CR and safety) along with corresponding 95% credible intervals, the Bayesian equivalent of 95% CIs. Ranking probabilities and surface under the cumulative ranking‐curve (SUCRA) were used to provide hierarchy probabilities. Highest SUCRA values (e.g., closer to 1) corresponded to a better position in the ranking of the treatment schedules. At the end of the analysis each of the treatment analyzed presented 5 different SUCRA scores, one for each endpoint. Beside ranking the treatments according to the mean of the different SUCRAs, we performed a dimensionality reduction through principal component analysis method in R (*prcomp* command) and grouped the treatments with the *cluster* package by an unsupervised automatic clustering according to similarities in outcomes results.^12^, ^13^ This allowed us to identify clusters of regimens with similar profiles of efficacy/safety rather than the “best” treatment.

## 3 RESULTS

### 3.1 Study selection and quality assessment

**FIGURE 1:**
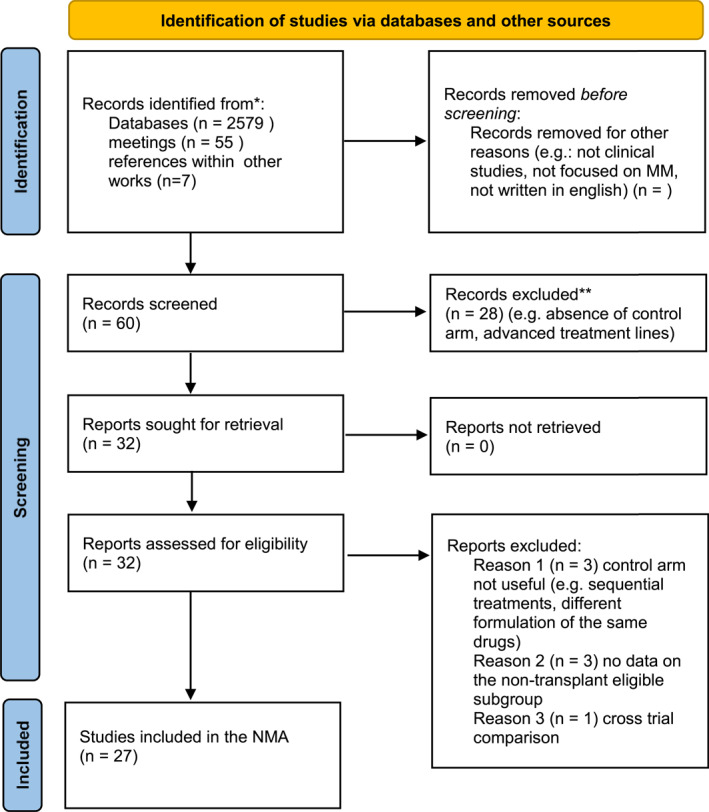
PRISMA (preferred reporting items for systematic reviews and meta‐analyses) flow chart reporting the whole work‐flow that lead to final study identification and selection

As shown in the PRISMA flow chart in Figure 1, with our search strategy we retrieved a total of 2579 studies. Of them, 27 studies, including a total of 12,935 patients were included in the final analysis (Table 1).^14^, ^15^, ^16^, ^17^, ^18^, ^19^, ^20^, ^21^, ^22^, ^23^, ^24^, ^25^, ^26^, ^27^, ^28^, ^29^, ^30^, ^31^, ^32^, ^33^, ^34^, ^35^, ^36^, ^37^, ^38^, ^39^, ^40^, ^41^, ^42^, ^43^, ^44^, ^45^ Almost all the trials included all the variables necessary to perform the whole analysis, and all the missing information where retrieved from other meta‐analysis, calculated from reported data, or obtained from updated analyses (e.g., OS data were often presented when a longer follow‐up was available).^46^, ^47^, ^48^, ^49^ All the trials selected presented data for PFS, OS, ORR, CR and safety analysis and were included in the NMA. In Supplementary Figure 1A are reported the data regarding the quality assessment: most of the study were reported as low risk in the majority of the evaluated criteria according to Cochrane guidelines. Additionally, the funnel plot in Supplementary Figure 1B confirmed the absence of publication biases.

**TABLE 1:** This table summarizes the main characteristics of all the studies included in the network meta‐analysis (NMA)

Trial	Year	Treatments	Patients	Most frequent G3‐4 AE
Facon/IFM 99‐06	2007	MPT/MP	321	Neutropenia
Palumbo	2008	MPTT/MP	331	Cytopenia
Hulin/IFM 01/01	2009	MPT/MP	229	Neutropenias
Waage	2009	MPTT/MP	357	Neutropenia
Ludwig	2009	TD/MP	288	Infections/Leukopenia
Beksac	2010	MPTT/MP	115	Cytopenia
Wijermans/Hovon49	2010	MPTT/MP	344	Infections
Mateos/Vista	2010	VMP/MP	682	Neutropenia
Palumbo	2010	VMPT/VMP	511	Neutropenia
Morgan/MRC myeloma IX	2011	CTD/MP	849	Cytopenia/Infections
Sacchi	2011	MPT/MP	118	Neutropenia
Palumbo/MM‐015	2012	MPRR/MPR/MP	459	Neutropenia
San Miguel	2013	VMPS/VMP	106	Neutropenia
Mateos/GEM2005	2014	VMP/VTP	260	Neutropenia
Hungria	2015	MPTT/TD/CTD	82	Neutropenia/Neuropathy
Keith Stewart/E1A06	2015	MPRR/MPTT	298	Neutropenia
Niesvizky/UPFRONT	2015	VD/VTD/VMP	502	Neuropathy
Magarotto	2016	MPR/CPR/RD9	662	Neutropenia
Zweegman	2016	MPRR/MPTT	637	Neutropenia
Durie/SWOGS0777	2016	VRD/RD	471	Neutropenia
Facon/FIRST	2018	MPT/RD/RD18	1623	Neutropenia/Infections
Mateos/ALCYONE	2018	VMPDr/VMP	706	Neutropenia
Facon/MAIA	2018	DrRD/RD	737	Neutropenia
Usmani/Keynote185	2018	PRD/RD	301	Neutropenia
Facon/CLARION	2019	KMP/VMP	955	Neutropenia
Facon/Tourmaline‐MM2	2021	IRD/RD	705	Neutropenia
Puig/CLARIDEX	2021	ClRD/RD	286	Infections

Abbreviations: ClRD, Clarithromycin + RD; CPR, Rd, cyclophosphamide/lenalidomide/prednisone; CTD, cyclophosphamide/thalidomide/dexamethasone; DrRd, Rd + Daratumumab; IRD, ixazomib + RD; KMP, Carfilzomib + MP; KMP, MP + carfilzomib; MP, melphalan/prednisone; MPT, MP + thalidomide; MPT‐T, MPT followed by thalidomide maintenance; MPR, MP + lenalidomide; MPR‐R, MPR followed by lenalidomide maintenance; PRd, Rd + pembrolizumab; TD, thalidomide/dexamethasone; VD, bortezomib/dexamethasone; VMP, Rd‐18, Rd for 18 months; Rd‐9, Rd for 9 months followed by R maintenance; VRD, VTD, VD + thalidomide; VRD, DrVMP, VMP + Daratumumab; VMPT, bortezomib‐melphalan‐prednisone‐thalidomide with bortezomib‐thalidomide maintenance; VMPS, VMP + siltuximab.

No significant inconsistency or loop‐specific heterogeneity were found in our NMA (data not shown).

### 3.2 Quadruplet and mAbs containing‐regimens consistently improve patients' outcome

**FIGURE 2:**
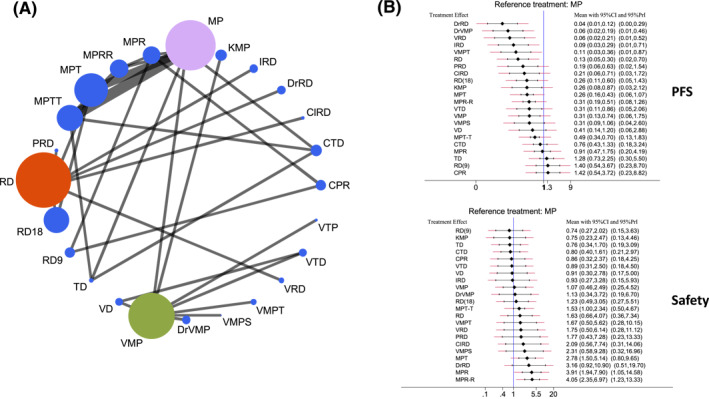
(A) Network plot of all treatment groups evaluated in the network meta‐analysis (NMA) for all the efficacy and safety endpoints. The size is proportional to the numbers of patients included in the analysis for each group and each connection represents the existence of direct comparisons data. (B) Effect estimates of the treatment in terms of progression‐free survival (PFS) and safety by using MP (melphalan prednisone) arm as comparator. Thalidomide/dexamethasone (TD), melphalan/prednisone (MP), bortezomib/dexamethasone (VD), Rd, cyclophosphamide/lenalidomide/prednisone (CPR), MP + thalidomide (MPT), MPT followed by thalidomide maintenance (MPT‐T), MP + lenalidomide (MPR), MPR followed by lenalidomide maintenance (MPR‐R), VMP, Rd for 18 months (Rd‐18), Rd for 9 months followed by R maintenance (Rd‐9), MP + carfilzomib (KMP), VRD, VD + thalidomide (VTD), cyclophosphamide/thalidomide/dexamethasone (CTD), VRD, VMP + Daratumumab (DrVMP), bortezomib‐melphalan‐prednisone‐thalidomide with bortezomib‐thalidomide maintenance (VMPT), Rd + Daratumumab (DrRd), VMP + siltuximab (VMPS), Rd + pembrolizumab (PRd), Carfilzomib + MP (KMP), Clarithromycin + RD (ClRD), ixazomib + RD (IRD)

Figure 2A shows the network of comparisons between all regimens evaluated. We identified a total of 23 different treatment arms/regimens (namely: thalidomide/dexamethasone (TD), melphalan/prednisone (MP), bortezomib/dexamethasone (VD), Rd, cyclophosphamide/lenalidomide/prednisone (CPR), MP + thalidomide (MPT), MPT followed by thalidomide maintenance (MPT‐T), MP + lenalidomide (MPR), MPR followed by lenalidomide maintenance (MPR‐R), VMP, Rd for 18 months (Rd18), Rd for 9 months followed by R maintenance (Rd9), MP + carfilzomib (KMP), VRD, VD + thalidomide (VTD), bortezomib/thalidomide/prednisone (VTP, which being part of the group of VT + steroids we aggregated with VTD) cyclophosphamide/thalidomide/dexamethasone (CTD), VMP + Daratumumab (DrVMP), bortezomib‐melphalan‐prednisone‐thalidomide with bortezomib‐thalidomide maintenance (VMPT), Rd + Daratumumab (DrRd), VMP + siltuximab (VMPS), Rd + pembrolizumab (PRd), Clarithromycin + RD (ClRD), ixazomib + RD (IRD)) to be compared (as reported in Table 1), linked by nine triangular loops.

Each group was subsequently compared against all other groups through a Bayesian NMA, and efficacy results for PFS and safety, using the MP regimen as comparator, are shown in Figure 1B in terms of HRs and credibility intervals (efficacy results in terms of OS, ORR, CR are shown in supplemental Figure 2A). Unsurprisingly, most modern regimens including DrRd, DrVMP and VRD, performed significantly better in terms of PFS as compared to all the other analyzed regimens, while RD(9) and CPR ranged among the worst regimens. Interestingly, DrRD, DrVMP, VRD, IRD, VMPT and RD reached a significant advantage against MP by using the most statistically restrictive “credibility intervals” from NMA. Similar results were obtained for the other efficacy endpoints with quadruplets regimens always reporting the better results (often reaching the statistical significance against MP) (Supplementary Figure 2A).

Regarding safety, regimens combining melphalan and lenalidomide delivered the highest toxicity to patients, while other regimens failed to demonstrate important differences.

### 3.3 DrRD and DrVMP could guarantee the best outcome for NEMM

**FIGURE 3:**
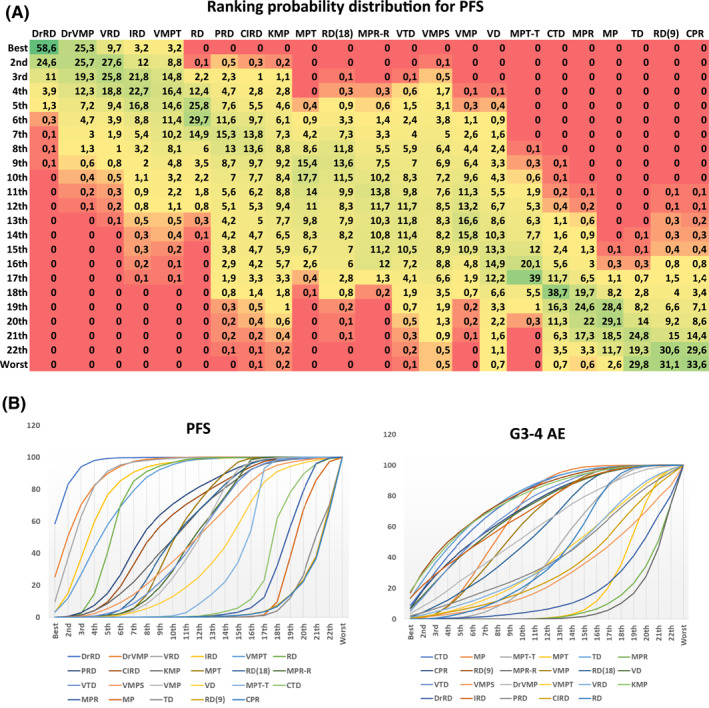
(A) Heatmap reporting the ranking probability of each regimen included in the meta‐analysis. The green color represents the highest probability of being in that position of the ranking chart, while the red represents the lowest probability. (B) Cumulative probability of being the *n*th in the ranking chart with respect to progression‐free survival (PFS) (left) or safety (right). The soonest the curve reaches the 100%, the highest is the probability of being better according to the endpoint analyzed

Network meta‐analysis has the possibility to calculate the probability of each regimen evaluated of being the best or the worst as well as the probable “position” within a ranking of all regimens. In Figure 3A the probability distribution of being the regimen placed at the “x” position in the PFS rank is showed. DrRd has a 58.6% probability of being the best regimen according to this outcome, immediately followed by DrVMP (25.3%) and VRD (9.7%). Figure 3B, which reports the cumulative probabilities, confirmed these results: indeed, in the “PFS” panel (left) the previously mentioned regimens were the first to reach the 100% cumulative probability, and were strongly separated from the other studied schedules. Regarding the safety panels (on the right), accordingly to what observed in the interval plots, no clear separation could be observed within this graph (all regimens reach the 100% cumulative probability in the late/right part of the graph) with the exception of melphalan/lenalidomide containing regimens, which were the worst schedules as demonstrated by the fact that were the last two reaching the top of the graph.

**FIGURE 4:**
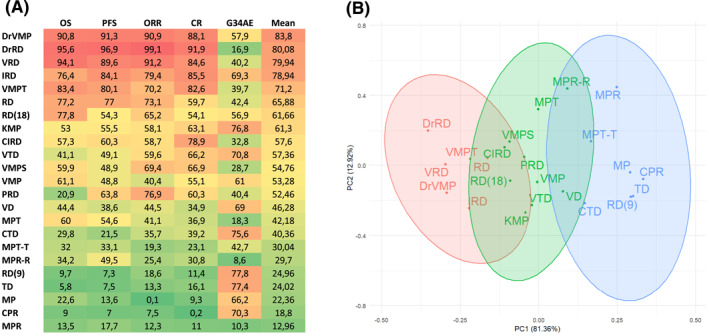
(A) Heatmap reporting the surface under the cumulative ranking‐curve (SUCRA) for each endpoint analyzed for each treatment schedule included in the analysis, ordered according to the mean SUCRA score (from the highest to lowest). (B) Principal component analysis reporting all the regimens analyzed grouped (unsupervised clustering) according to their SUCRA profile (the most similar are the SUCRA scores for each endpoint, the closest are the schedules within the picture). Thalidomide/dexamethasone (TD), melphalan/prednisone (MP), bortezomib/dexamethasone (VD), Rd, cyclophosphamide/lenalidomide/prednisone (CPR), MP + thalidomide (MPT), MPT followed by thalidomide maintenance (MPT‐T), MP + lenalidomide (MPR), MPR followed by lenalidomide maintenance (MPR‐R), VMP, Rd for 18 months (Rd‐18), Rd for 9 months followed by R maintenance (Rd‐9), MP + carfilzomib (KMP), VRD, VD + thalidomide (VTD), cyclophosphamide/thalidomide/dexamethasone (CTD), VRD, VMP + Daratumumab (DrVMP), bortezomib‐melphalan‐prednisone‐thalidomide with bortezomib‐thalidomide maintenance (VMPT), Rd + Daratumumab (DrRd), VMP + siltuximab (VMPS), Rd + pembrolizumab (PRd), Carfilzomib + MP (KMP), Clarithromycin + RD (ClRD), ixazomib + RD (IRD)

Finally, we investigated which regimen, among all regimens included in the NMA, scores as the overall best regimen. To find this answer, we determined the SUCRA values for PFS, OS, ORR, CR, and safety and estimated an average value to rank all the treatments options included in our analysis (Figure 4A). According to average SUCRA values, the DrVMP regimen achieved the highest score (average SUCRA: 83.8) closely followed by DrRd (80.08) (which is better than DrVMP in every field with the exception of safety), VRD (79.94) and IRD (78.94). It should be noted that the top two regimens were Daratumumab based triplets, and that three out of five top regimens are based on Rd backbone.

### 3.4 PCA analysis identified the best regimens according to needed outcomes

To overcome the limit of using a simple and not weighted “average” of the SUCRA score, we applied a dimensionality reduction approach known as “principal component analysis,” PCA, to distribute in a plane all the 23 evaluated regimens. The distance between each point depends upon the difference in the “profile” of SUCRA scores. By using this approach we were able to unbiasedly cluster all the evaluated regimens into three different groups (Figure 4B and Supplementary Figure 2B): (1) DrRd, DrVMP, VRD, IRD, VMPT and Rd as the preferred regimens to be used for first line approach (the most important determinants of this group were all the efficacy outcome as reported in Supplementary Figure 2B); of note, DrRd appears to be separated from other regimens (maybe due to the better results obtained in all the efficacy endpoints), while DrVMP and VRD are very close, underscoring the similarity of outcome obtained with both regimens; (2) 10 regimens (VMP, VTD, VD, Rd(18), MPR_R, MPT, VMPS, ClRD, PRD, KMP) to be considered as potentially alternative regimens when the ones of first group are not available; and (3) seven regimens (MPR, MPT‐T, MP, CPR, TD, RD(9), CTD) with the lowest probability of being beneficial in frontline.

### 3.5 MRD assessment further support NMA results

Currently, the absence of detectable minimal residual disease (MRD), especially if sustained, is considered the best surrogate marker of OS.^50^ Along this line we retrieved the rates of MRD negativity in each study that investigated/disclosed this endpoint. Unfortunately, 4 studies only reported these results (Table 2). Interestingly, both DaraRD and DaraVMP reported similar MRD negativity rates, a result that further supports the conclusion of our NMA. No data regarding the SWOG5077, and specifically, the VRD regimen, were reported in any other study on NEMM patients.

**TABLE 2:** The methodologies and the results of minimal residual disease (MRD) determination are reported in this table

	Treatments	MRD undetectable	Method
ALCYONE	DrVMP versus VMP	28% versus 7%	Adaptive Biotechnologies clonoSEQ assay
MAIA	DrRD versus RD	24.2% versus 7.3%	Adaptive Biotechnologies clonoSEQ assay
CLARION	KMP versus VMP	7.9% versus 7.8%*	NGF
CLARIDEX	ClRD versus RD	2.8% versus 3.5%*	NGF

*Note*: Unfortunately for 4 trials only these results are available.

Abbreviations: MRD, minimal residual disease; NGF, next generation flow cytometry.

## 4 DISCUSSION

The landscape of first line treatment for NEMM has dramatically changed over the past 20 years.^2^, ^4^ Starting from the introduction of the first PIs and IMiDs, the increase in the knowledge of immunological and biological determinants of myeloma evolution,^3^, ^4^, ^51^, ^52^, ^53^ enriched the clinical scenario of new schedules and molecules, including the recently approved monoclonal antibodies (the anti‐CD38 daratumumab and isatuximab). Unfortunately, the lack of head‐to‐head comparisons between the regimens considered as standard of care, complicates the therapeutic decision making. On these bases, the aim of our study was to systematically review and compare the activity and safety of new regimens including three or four drugs as well as novel agents such as mAbs, investigated in NEMM since the introduction of PIs or IMiDs. To this end, we believe that Bayesian NMAs are the best tool for exploring the strength of evidence for regimens that have not undergone direct comparison.^10^ Indeed, this NMA by ranking treatments according to several activity and safety markers, could facilitate the decision making in the transplant‐ineligible MM setting, taking into account that the “clinical” environment (including patients' willingness) should be carefully considered before treatment selection. Accordingly, we demonstrated, by merging the results of 27 different trials, that regimens including daratumumab perform better in term of every efficacy endpoints, bringing an acceptable safety profile, a result further underscored by ranking regimens according to the “average” SUCRA score. Interestingly, three out of four of the “better” regimens were triplets including the Rd backbone plus a PI or a mAb. Surprisingly, while performing better in each efficacy endpoint, the overall mean SUCRA of DrRd was lower than the one achieved by the quadruplet DrVMP (80.08 vs. 83.8, respectively). This latter point underline a major limitation of NMA: this approach could rank treatments according to one specific end‐point only, and an “average” score, by mixing results obtained in different aspects, could not be able to capture the overall efficacy/safety profile of a regimen.^5^ On these bases, we used a dimensional reduction approach (principal component analysis) and the k‐means derived algorithm partitioning around medoids to group the different treatments according to their efficacy and safety profiles.^13^ Therefore, we obtained three groups: one efficacy‐driven group, a second “alternative” group and a third “bad” group which includes schedules considered neither the safest nor the most effective. On these bases we considered DrRD, DrVMP and VRD as the preferred regimens to be used in NEMM, with the option to consider VMPT, IRD or even the doublet Rd as reasonable alternatives. Among the alternative regimens, VMP, KMP or even the double VD could be still considered for selected patients. These results have a substantial relevance in the decision‐making algorithm for the treatment of these patients, especially if we take into account that DrRd regimen is not the absolute “winner”. Indeed, the choice between the Dara‐containing regimens or the VRD triplet should take into account different points: (1) according to the registrative clinical trials, the median PFS were about 60, 36 and 41 months for DrRd, DrVMP and VRD respectively^18^, ^19^, ^20^, ^21^, ^54^; of note, the long PFS registered for VRD within the SWOG5077 trial is affected by the high percentage of transplant eligible patients enrolled in the trial, nevertheless, we decided to include it in the whole analysis due to the fact that this schedule is currently approved in the NEMM setting based on the results of this trial. However, the PFS estimation of 35 months, observed in a recent phase 2 study exploring a modified VRD combination for NEMM, potentially represents a more realistic result.^55^ (2) No clear differences could still be observed in OS between the 3 regimens^18^, ^19^, ^20^, ^21^, ^54^; this event could be due to the fact that the appearance of lenalidomide resistance reduces the PFS2 of MM patients,^5^ negatively affecting OS of these groups, or by the fact that subsequent treatment lines could compensate the initial difference among these regimens. (3) The achievement of an (sustained) undetectable MRD state is considered the best surrogate marker of OS.^50^ Accordingly, both DrRd and DrVMP, while reporting notable differences in term of PFS (but still not in os), achieved similar rates of undetectable MRD, a result in line with the conclusion of our NMA. Furthermore, a recent study on pooled patients from MAIA and ALCYONE trials demonstrated that daratumumab significantly increases the probability of achieving a sustained (>12 months) MRD negativity status and that this significantly improves both PFS and PFS2. Interestingly, despite obtaining a higher percentage of MRD negativity at 12 months (14 vs. 10.9%, DrVMP vs. DrRd respectively), MM patients treated with a (quite) fixed duration treatment (DrVMP/VMP) lose the “long time” effect which could be observed with the continuous lenalidomide‐based regimens (DrRd/RD) (at the price of an increased overall toxicity), while retaining the advantage of a better PFS2.^56^ It is therefore of utmost importance, to discuss with the patients about schedule‐specific administrations rules. Indeed, lenalidomide and Daratumumab are administered until disease progression while bortezomib is discontinued after nine treatment courses in DrVMP and after 15 cycles in VRD lite (or eight cycles in VRD standard).^55^

Currently, no data about the possible best “sequencing” options are available. Additionally, due to the unavoidable increase in the use of Daratumumab‐based regimens, most patients will be daratumumab/lenalidomide double refractory at the beginning of second line of treatment, thus representing an emerging medical need. Taking into account that we have no data on the possibility of continuing the treatment with an anti‐CD38 mAb after progression (we could consider isatuximab‐based combinations after holding the anti‐CD38 for one line, i.e., we should wait a second relapse), pomalidomide/bortezomib/dexamethasone or carfilzomib/dexamethasone combinations are the best therapeutic options for these patients.^1^, ^5^ On the other side, for patients progressing after a DrVMP regimen, the combination of carfilzomib, lenalidomide and dexamethasone represent a valuable option.^1^ On these bases, we could start to imagine a chemo‐free treatment history for myeloma patients, where immunotherapy (IMiDs, bispecific agents, CAR‐T)^57^ as well as drugs able to elicit a strong autologous immune response (immunogenic cell death inducers, such as bortezomib or innovative target drugs such as STING agonists, hypomethylating agents or cancer vaccines)^52^ will be combined to achieve long and sustained responses with minimal toxicities.

In the last 10 years several NMA in this field have been published,^46^, ^47^, ^58^, ^59^, ^60^, ^61^, ^62^ each of them with its own limitations which reflect the fact that this method could not completely replace a randomized clinical trial. Anyway, the most recent ones are in line with the results of our NMA, where the addition of Daratumumab to the previous standard‐of‐care RD and VMP should be considered as the preferred regimen in NEMM, a result further supported by the achievement of similar results in term of MRD negativity.^50^

Our work presents some limitations that should be carefully taken into account: first, all data were retrieved or calculated from published studies rather than from individual patients'; second, potential biases can be produced by the heterogeneity of the agents, patient populations as well as the long timeframe included in the analysis: to reduce this factor, we tried to limit the timeframe to the latest 20 years, that is, from the introduction of modern drugs (IMiDs and PIs). Finally, this work should be considered a snapshot of current evidence that could quickly evolves with the introduction of new drugs in the frontline setting.

## 5 CONCLUSION

Overall, this is, to our knowledge, the first NMA which use a dimensionality reduction approach to group treatments according to their efficacy/safety profiles, thus overcoming the limitation of NMA of being endpoint specific. Finally, our work supports a multiparametric approach in the decision‐making of the first line therapy for NEMM patients: indeed, while the updated results of MAIA trial showed impressive results in term of PFS for the DrRd combination, our results demonstrated a substantial evidence‐based overlap between Daratumumab‐based regimens (DrRd/DaraVMP: no differences in term of OS/MRD) and further support the use of VRD (especially for less fit patients) for the frontline treatment of NEMM patients.

## AUTHOR CONTRIBUTIONS

Cirino Botta conceived and designed the study; Cirino Botta, Emilia Gigliotta, Rita Anselmo and Marco Santoro acquired and revised the data; Sergio Siragusa, Jesus San Miguel, Massimo Gentile, Pierpaolo Correale and Bruno Paiva supervised the study; Cirino Botta, Emilia Gigliotta and Marco Santoro did the statistical analysis; Paula Rodriguez Otero, Melania Carlisi, Concetta Conticello, Alessandra Romano, Antonio Giovanni Solimando, Claudio Cerchione, Matteo Da Vià, Francesco Di Raimondo and Niccolò Bolli read different drafts over the development of the whole work giving important analytical suggestions which have been necessary for reaching the final results; Cirino Botta, Emilia Gigliotta and Sergio Siragusa wrote the final draft. All authors read and approved the final draft.

## CONFLICT OF INTEREST

The author declares they have no conflict of interest.

### TRANSPARENT PEER REVIEW

The peer review history for this article is available at https://publons.com/publon/10.1002/hon.3041.

## Supporting information

Supporting Information S1

## Data Availability

Data sharing not applicable to this article as no datasets were generated or analyzed during the current study.
