# Supplementary figures and images for: Network meta‐analysis of randomized trials in multiple myeloma: Efficacy and safety in frontline therapy for patients not eligible for transplant
Source: Hematol Oncol. 2022 Jul 11;40(5):987–98. doi: 10.1002/hon.3041 (PMC10084226; doi:10.1002/hon.3041)

## Slide 1
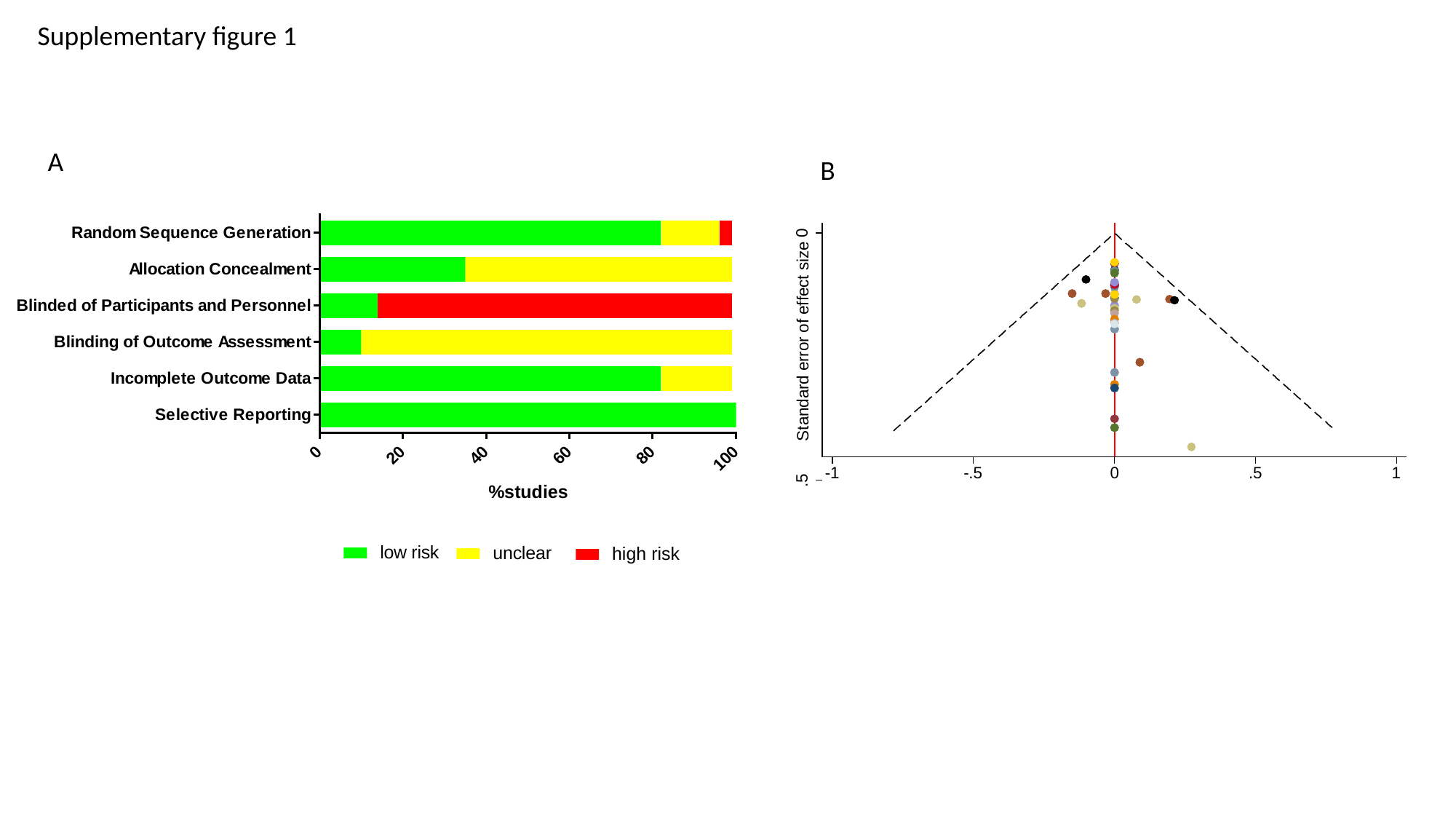

Supplementary figure 1
A
B

## Slide 2
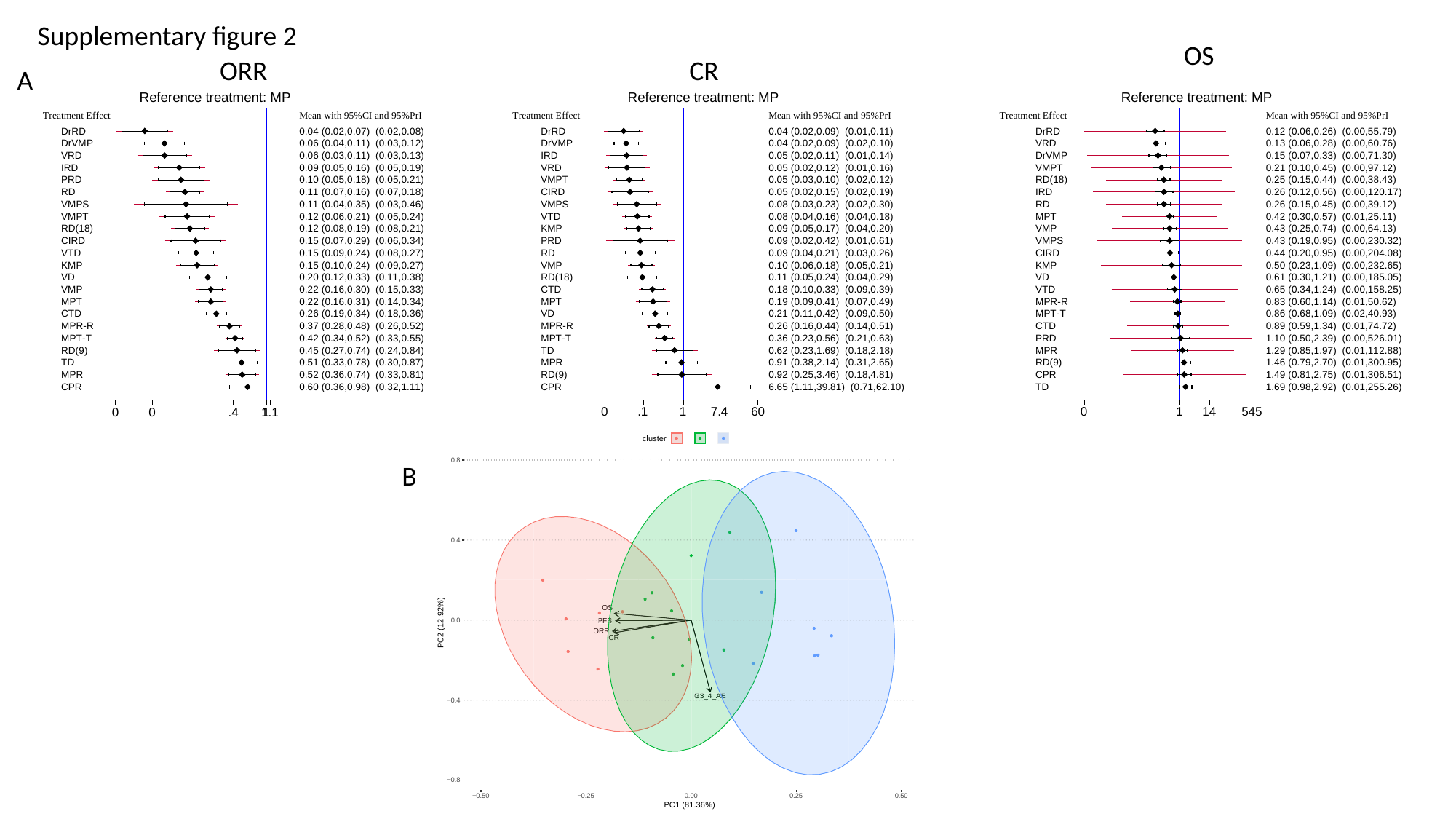

Supplementary figure 2
OS
ORR
CR
A
B

Supplement: Supplementary file 1 — Supporting Information S1 [file HON-40-987-s001.pptx]
